# An Anisotropic Auxetic 2D Metamaterial Based on Sliding Microstructural Mechanism

**DOI:** 10.3390/ma12030429

**Published:** 2019-01-30

**Authors:** Teik-Cheng Lim

**Affiliations:** School of Science and Technology, Singapore University of Social Sciences, Singapore 599494, Singapore; tclim@suss.edu.sg; Tel.: +65-62489252

**Keywords:** anisotropic, auxetic, metamaterials, microstructure, slot and slider mechanism

## Abstract

A new 2D microstructure is proposed herein in the form of rigid unit cells, each taking the form of a cross with two opposing crossbars forming slots and the other two opposing crossbars forming sliders. The unit cells in the microstructure are arranged in a rectangular array in which the nearest four neighboring cells are rotated by 90° such that a slider in each unit cell is connected to a slot from its nearest neighbor. Using a kinematics approach, the Poisson’s ratio along the axes of symmetry can be obtained, while the off-axis Poisson’s ratio is obtained using Mohr’s circle. In the special case of a square array, the results show that the Poisson’s ratio varies between 0 (for loading parallel to the axes) and −1 (for loading at 45° from the axes). For a rectangular array, the Poisson’s ratio varies from 0 (for loading along the axes) to a value more negative than −1. The obtained results suggest the proposed microstructure is useful for designing materials that permit rapid change in Poisson’s ratio for angular change.

## 1. Introduction

Auxetic materials are solids that exhibit a negative Poisson’s ratio, i.e., such materials expand laterally when stretched axially and contract laterally when compressed axially and are therefore classified under the broad category of metamaterials with negative properties. Metamaterials are materials that are engineered to possess characteristics that are not exhibited in naturally occurring materials; they consist of smaller units arranged in such a manner that the metamaterial behavior arises from the geometrical microstructures rather than from that of the base material. Research in auxetic materials began in earnest with the works of Lakes [1,2] and Wojciechowski et al. [3,4,5], leading to a great number of potential applications [6,7,8,9,10,11,12,13,14,15,16,17,18,19,20,21,22,23]. Due to the negativity of Poisson’s ratio, auxetic fibers are resistant towards pull-out from the matrix material because the tensile load of the fiber causes the fiber diameter to increase, thereby producing a self-locking mechanism. Auxetic sheets are suitable for wrapping around dome-shaped surfaces because the action of bending on two opposing sides of the sheet material causes the other two sides to curve in similar way to form a synclastic shape. In addition, an auxetic half-space is useful for reinforcing against projectile impact because the action of point load on a surface causes the material to move radially towards the line of force. Pertaining to the last example, Shodja et al. [24] showed a very strong influence of the Poisson’s ratio, via the elastic constants, on Boussinesq indentation of a transversely isotropic half-space embedded with an inextensible membrane. 

The application of auxetic materials vis-à-vis conventional ones is of interest as the overall properties of structures made of auxetic materials differ from—and, under some circumstances, are not achievable by—structures made from conventional ones. By way of example, a study of the axisymmetric response of a bi-material full-space with an interfacial thin film by Ahmadi et al. [25] showed that in a reinforced homogeneous full-space made from materials with small positive Poisson’s ratio or with negative Poisson’s ratio, the thin film reinforcement has an insignificant influence on elastic responses. In addition, reinforced full-spaces made of auxetic materials exhibit more compliant properties in comparison to reinforced conventional materials [25].

Of late, progress has been made on planar tessellations that are capable of controlling the Poisson’s ratio, including triggering auxetic behavior [26,27]. In addition to the microstructural level, auxeticity can also be derived by introducing perforations [28] and other forms of porosity [29,30]. Due to an exponential increase in this area of research in the past decades, it is no longer possible to list relevant works sufficiently. The reader is referred to recent comprehensive reviews in auxetic materials [31,32,33,34,35,36] and a related monograph [37]. The proposed microstructure resembles the interlocking hexagons model [38], in which no microstructural rotation occurs, but, instead, sliding takes place between neighboring blocks. Essentially, the unit cells are rigid or of a very high stiffness such that the assumption of rigid units is valid and only translational motion takes place. One such example has been suggested by Alderson and Scarpa [39] in regard to eliminating mechanical vibrations and noise experienced by newborn babies during transfer in vehicles, whereby the interlocking model could be used to help make safer neonatal transfer vehicles. 

**Figure 1:**
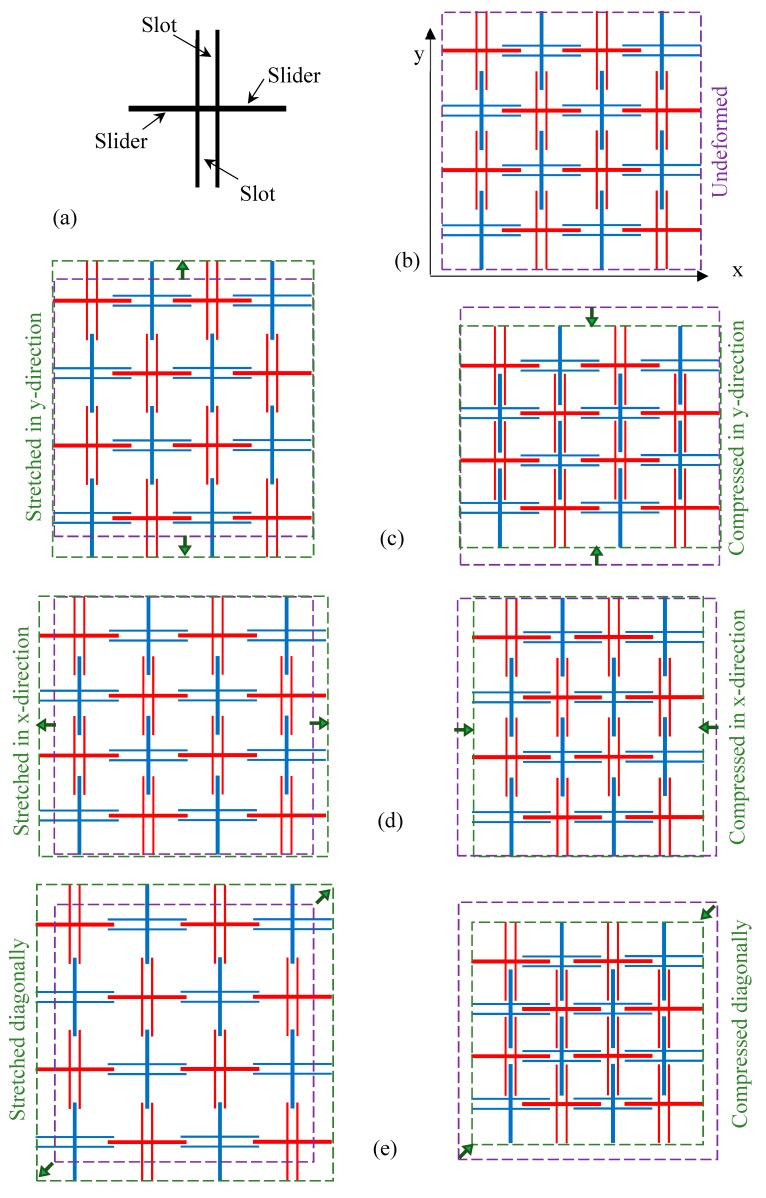
(**a**) A unit cell of the microstructure; (**b**) 4 × 4 unit cells in the original state; (**c**) loading in the *y* direction; (**d**) loading in the *x* direction; and (**e**) off-axis loading. Note: the dashed purple squares indicate the undeformed boundaries, while the dashed green squares or rectangles denote deformed boundaries.

In this paper, a 2D metamaterial is proposed to exhibit zero Poisson’s ratio along its principal axes, while exhibiting negative values of Poisson’s ratio in other directions, i.e., the microstructure exhibits great sensitivity in change in Poisson’s ratio from change in loading direction. The proposed 2D metamaterial and some of its deformation mechanisms are illustrated in Figure 1. Each unit cell is rigid and takes the form of a cross such that two opposing crossbars are slots and the other two opposing crossbars are sliders, as shown in Figure 1a. The unit cells in the microstructure are arranged in a rectangular array with the nearest four neighboring cells rotated by 90° so that a slider from one cell is placed in the slot of its neighbor. A sample of 4 × 4 unit cells that make up the microstructure is furnished in Figure 1b. This indicates the microstructural conformation at the original, or undeformed, state; a dashed purple square that encompasses the given microstructure before deformation is imposed on the microstructure with deformation so as to facilitate comparison against the former. Under loading along the *y* direction, as shown in Figure 1c, the Poisson’s ratio is *v_yx_* = 0, while loading in the direction of the *x* axis, as shown in Figure 1d, gives *v_xy_* = 0. Under the action of off-axis loading, as indicated in Figure 1e, we have a negative Poisson’s ratio. The underlying hypothesis requires that the relative motion of each unit, with reference to its neighboring unit cell, is uniform. It follows that the increase or decrease in the gap is uniform when measured from each axis. It is further assumed that the contact areas between the slot and slider are frictionless, or that the effect of friction is negligible, as a result of sufficient lubrication. With the advancement of rapid prototyping technology, the currently proposed microstructure can be designed and fabricated using 3D printing or kirigami manufacture.

## 2. Analysis

**Figure 2:**
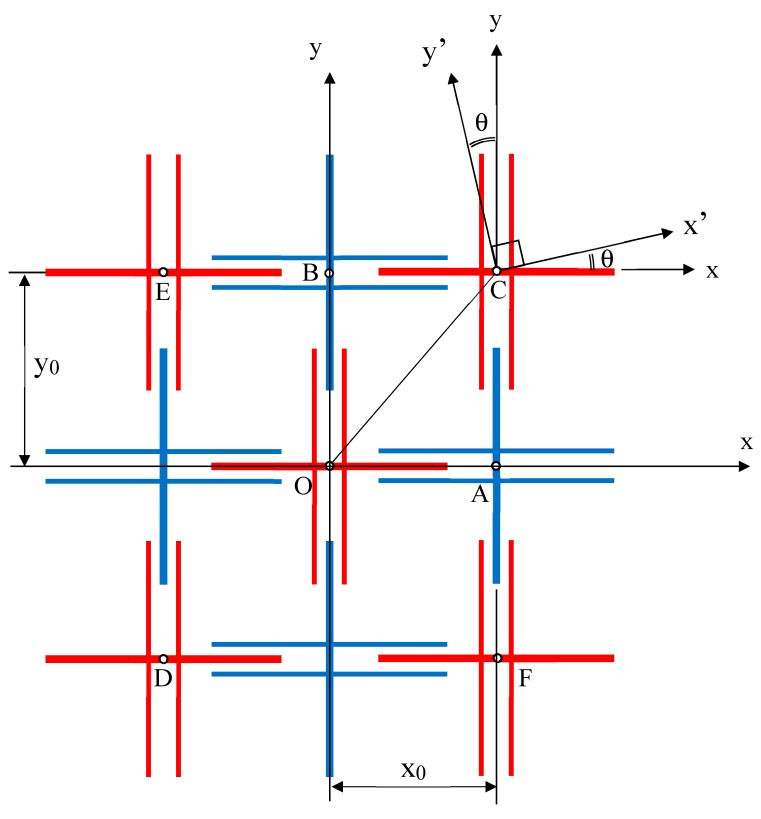
Schematic view for analysis.

The analysis of Poisson’s ratio for the proposed 2D metamaterial is made in reference to Figure 2, wherein neighboring unit cells are spaced at distances of *x*_0_ and *y*_0_ along the *x* axis and *y* axis, respectively. While the analysis of Poisson’s ratio values *v_xy_* and *v_yx_* can be easily made by sliding cell A along the *x* axis and by sliding cell B along the *y* axis, such an approach does not permit the analysis of the Poisson’s ratio in the other direction. Hence, a general approach can be attempted by taking the displacement of cell C with respect to cell O, which the origin of the coordinate system lies on. Let θ indicate the loading direction. By symmetry, it is sufficient to model the movement of C for 0° < θ < 90°. The movement of C in the direction 90° < θ < 180° is not defined as it would be appropriate to model the displacement of cell E in that direction with reference to cell O. Similarly, the motion of C in the direction 270° < θ < 360° is undefined, as it would be proper to do so for cell F in that direction with reference to cell O. The displacement of C in the direction 180° < θ < 270° indicates compressive loading, but this is not required as the analysis of loading in the 0° < θ < 90° direction includes that in the 180° < θ < 270° direction by using negative values for the displacement components. 

**Figure A1:**
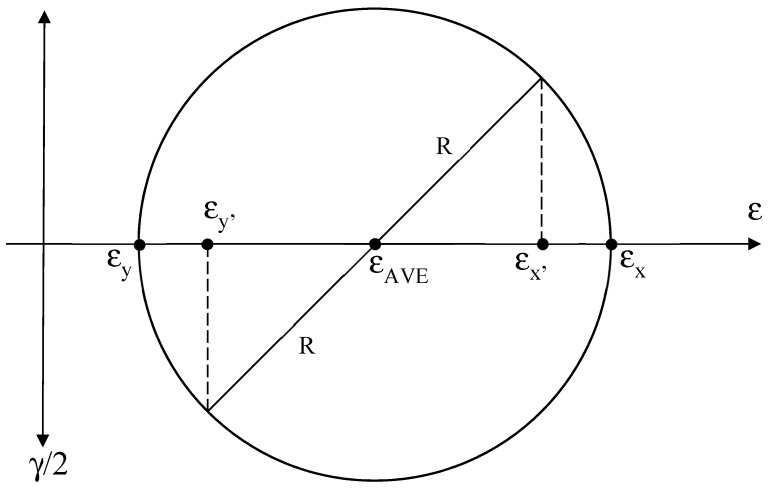
Obtaining the strains parallel and perpendicular to loading direction (*x*’,*y*’) from the strains along the axes of symmetry (*x*,*y*).

To cater for off-axis loading, we introduce on the cell C the local *x*’–*y*’ axes, which are rotated by an angle of θ anti-clockwise from the global *x*–*y* axes, such that the *x*’ axis coincides with the loading direction. Let cell C displace by a distance *dx’*. Resolving the d*x*’ displacement along the *x* axis and the *y* axis, we have

(1) $$\begin{array}{c} dx=dx\prime \cos\theta \\ dy=dx\prime \sin\theta \end{array}$$

which lead to the corresponding strains

(2) $$\begin{array}{c} \varepsilon_{x}=\frac{dx}{x_{0}}=\frac{dx\prime }{x_{0}}\cos\theta \\ \varepsilon_{y}=\frac{dy}{y_{0}}=\frac{dx\prime }{y_{0}}\sin\theta \end{array}$$

in the *x* axis and *y* axis, respectively. Since the *x* and *y* axes are axes of symmetry, the corresponding strains are principal strains. Upon recognizing that Equation (2) gives the principal strains, the strains in the *x*’ and *y*’ directions can be obtained using Mohr’s circle for strain, as shown in the Appendix A. From Figure A1 of the Appendix A, we have

(3) $$\varepsilon_{AVE}=\frac{\varepsilon_{x}+\varepsilon_{y}}{2}=\frac{dx\prime }{2}\left(\frac{\cos\theta }{x_{0}}+\frac{\sin\theta }{y_{0}}\right)$$

and

(4) $$R=\frac{\varepsilon_{x}-\varepsilon_{y}}{2}=\frac{dx\prime }{2}\left(\frac{\cos\theta }{x_{0}}-\frac{\sin\theta }{y_{0}}\right)$$

which lead to the strains in the direction of loading *x*’ of

(5) $$\varepsilon_{x\prime }=\varepsilon_{AVE}+R\cos2\theta =\frac{dx\prime }{2}\left[\frac{\cos\theta }{x_{0}}\left(1+\cos2\theta \right)+\frac{\sin\theta }{y_{0}}\left(1-\cos2\theta \right)\right]$$

and in the direction perpendicular to the direction of loading of

(6) $$\varepsilon_{y\prime }=\varepsilon_{AVE}-R\cos2\theta =\frac{dx\prime }{2}\left[\frac{\cos\theta }{x_{0}}\left(1-\cos2\theta \right)+\frac{\sin\theta }{y_{0}}\left(1+\cos2\theta \right)\right]$$

The off-axis Poisson’s ratio, *v_x’y’_*, is therefore

(7) $$v_{x\prime y\prime }=-\frac{\varepsilon_{y\prime }}{\varepsilon_{x\prime }}=-\frac{\frac{\cos\theta }{x_{0}}\left(1-\cos2\theta \right)+\frac{\sin\theta }{y_{0}}\left(1+\cos2\theta \right)}{\frac{\cos\theta }{x_{0}}\left(1+\cos2\theta \right)+\frac{\sin\theta }{y_{0}}\left(1-\cos2\theta \right)}$$

It can be seen that the substitution of θ = 0° (i.e., *v_x’y’_* = *v_xy_*) into Equation (7) gives *v_x’y’_* = 0, and likewise the substitution of θ = 90° (i.e., *v_x’y’_* = *v_yx_*) into Equation (7) also gives *v_x’y’_* = 0. The Poisson’s ratio results for off-axis loading are discussed in the next section.

## 3. Results and Discussion

If the cells are arranged such that OC makes an angle of 30° with the *x* axis (i.e., *y*_0_ = *x*_0_/√3) or 60° with the *x* axis (i.e., *y*_0_ = *x*_0_√3), then the off-axis Poisson’s ratio equations simplify to

(8) $$v_{x\prime y\prime }=-\frac{\cos\theta \left(1-\cos2\theta \right)+\sqrt{3}\sin\theta \left(1+\cos2\theta \right)}{\cos\theta \left(1+\cos2\theta \right)+\sqrt{3}\sin\theta \left(1-\cos2\theta \right)}$$

and

(9) $$v_{x\prime y\prime }=-\frac{\sqrt{3}\cos\theta \left(1-\cos2\theta \right)+\sin\theta \left(1+\cos2\theta \right)}{\sqrt{3}\cos\theta \left(1+\cos2\theta \right)+\sin\theta \left(1-\cos2\theta \right)}$$

respectively. For the special case of a square array (i.e., *y*_0_ = *x*_0_), the Poisson’s ratio is further reduced to

(10) $$v_{x\prime y\prime }=-\frac{\cos\theta \left(1-\cos2\theta \right)+\sin\theta \left(1+\cos2\theta \right)}{\cos\theta \left(1+\cos2\theta \right)+\sin\theta \left(1-\cos2\theta \right)}$$

**Figure 3:**
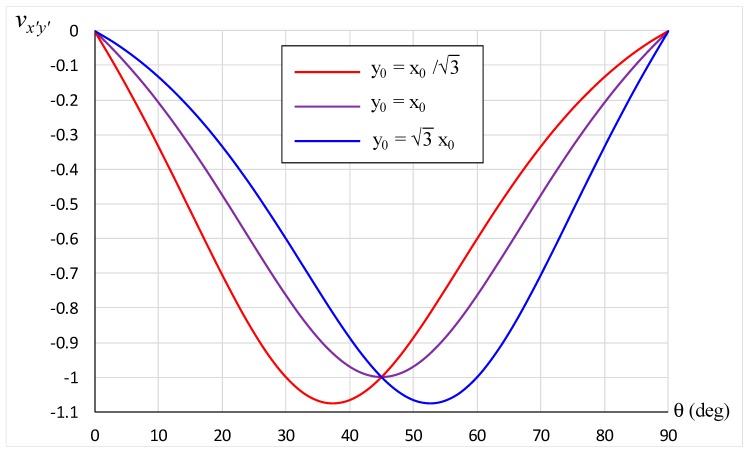
Variation of Poisson’s ratio with loading direction for a square array $y_{0}=x_{0}$ (purple) and for rectangular arrays with $y_{0}=\sqrt{3}x_{0}$ (blue) and $y_{0}=x_{0}/\sqrt{3}$ (red).

The variation of the off-axis Poisson’s ratio with the direction of loading for these three spatial arrays is plotted in Figure 3. It can be seen that the most negative Poisson’s ratio for a square array occurs when loading is imposed in the diagonal direction, with a value of −1. A greater extent of auxeticity is obtained when the cell array is not square (*y*_0_ ≠ *x*_0_). Specifically, the minimum *v_x’y’_* for a rectangular array is more negative than −1 due to the highly anisotropic nature of this microstructural mechanism.

Particular case studies can be made for the situation wherein the loading direction is parallel to OC in Figure 2. This occurs when the unit cells in the first and third quadrants are stretched away from, or compressed towards, the origin. A similar effect is obtained when the unit cells in the second and fourth quadrants are loaded such that the line of force passes through O. Under such a category, we note that

(11) $$\tan\theta =\frac{y_{0}}{x_{0}}$$

**Figure 4:**
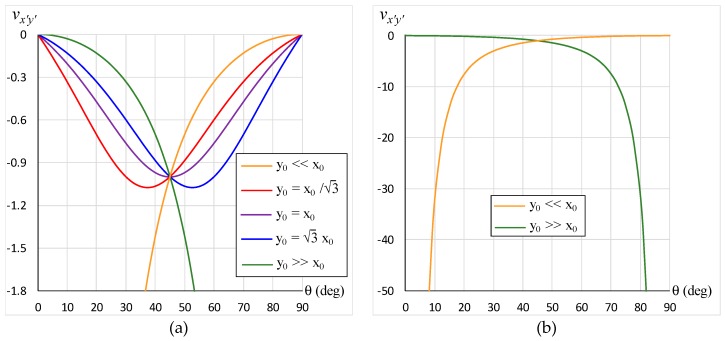
Variation of Poisson’s ratio with loading direction for extreme arrays (yellow and green): (**a**) in comparison to moderate arrays (red, purple and blue), and (**b**) a visual display on the extent of auxeticity.

Substituting Equation (11) into Equation (7) gives *v_x’y’_* = −1. This result is displayed in Figure 3 for θ = 30°, 45°, and 60° when *y*_0_/*x*_0_ = tan 30°, *y*_0_/*x*_0_ = tan 45°, and *y*_0_/*x*_0_ = tan 60°, respectively. Another set of particular cases takes place when the array is extreme, such that Equation (7) simplifies to

(12) $$v_{x\prime y\prime }=-\frac{1\pm \cos2\theta }{1\mp \cos2\theta }$$

where the upper and lower signs correspond to *y*_0_ << *x*_0_ and *y*_0_ >> *x*_0_, respectively. This is shown in Figure 4a to facilitate comparison between the Poisson’s ratio for extreme arrays (*y*_0_ << *x*_0_ and *y*_0_ >> *x*_0_) with that for moderate arrays ($y_{0}=x_{0}$, $y_{0}=\sqrt{3}x_{0}$, and $y_{0}=x_{0}/\sqrt{3}$). Figure 4b demonstrates the manner in which the Poisson’s ratio changes as the loading direction approaches the axes. 

**Figure 5:**
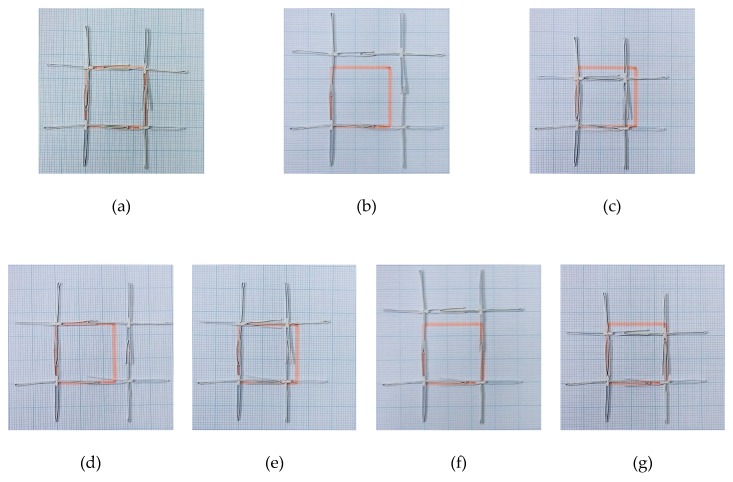
Proof-of-concept demonstration for the currently described sliding microstructural mechanism using four unit cells: (**a**) *ε_x_* = *ε_y_* = 0, (**b**) *ε_x_* = *ε_y_* = 0.2, (**c**) *ε_x_* = *ε_y_* = −0.2, (**d**) *ε_x_* = 0.2, *ε_y_* = 0, (**e**) *ε_x_* = −0.2, *ε_y_* = 0, (**f**) *ε_x_* = 0, *ε_y_* = 0.2, and (**g**) *ε_x_* = 0, *ε_y_* = −0.2. An orange-colored square indicates the area formed by the center of the four unit cells before deformation (**a**) to facilitate comparison with (**b**) to (**g**).

As the current model assumes rigid unit cells, one may expect the actual properties to deviate if the deformation of the unit cells is significant. Specifically, the flexure of the slots and sliders as beam deflections would reduce the strains perpendicular to the direction of the deflected slots and sliders. However, with sufficient lubrication between the slot of a unit cell and the slider in a neighboring unit cell, the suggested microstructure permits smooth relative motion between neighboring unit cells with insignificant deformation of the unit cells. Evidence of this observation is furnished in Figure 5 with a square array (*x*_0_ = *y*_0_) for (a) the original state, as well as for (b) stretching and (c) compression along θ = 45°, while stretching and compression along the axes are shown in Figure 5d–g.

## 4. Conclusions

While most auxetic microstructures exhibit some form of rotation, with the exception of the interlocking hexagons model [38], a new microstructure that is based on sliding mechanism is proposed herein. Unlike the interlocking hexagons model, the currently proposed model exhibits a high level of porosity, and therefore more suitable for applications where low weight is desired. In general, the model demonstrates two axes of symmetry, but the axes of symmetry increase to four in the case of a square array. For the special case of a square array, the Poisson’s ratio fluctuates between 0 and −1 for every 45° change in loading direction. A greater extent of auxeticity can be found when the array is not square. This is due to the high level of anisotropy of the microstructure. The extent of anisotropy increases when the array goes to the extremes, thereby leading to extreme negative Poisson’s ratios. The results from this paper, in conjunction with earlier works on auxetic beams [40,41], auxetic rods [42,43,44], auxetic plates [45,46,47,48,49,50,51,52], auxetic shells [53,54], auxetic composites [55,56,57,58,59,60,61,62] and 2D metamaterial structures [63,64], would avail more design options for the engineer in developing novel load bearing materials and structures.
